# Maternal perinatal and concurrent depressive symptoms and child behavior problems: a sibling comparison study

**DOI:** 10.1111/jcpp.12704

**Published:** 2017-02-23

**Authors:** Line C. Gjerde, Espen Moen Eilertsen, Ted Reichborn‐Kjennerud, Tom A. McAdams, Henrik Daae Zachrisson, Imac Maria Zambrana, Espen Røysamb, Kenneth S. Kendler, Eivind Ystrom

**Affiliations:** ^1^ Department of Mental DisordersNorwegian Institute of Public HealthOsloNorway; ^2^ Department of PsychologyUniversity of OsloOsloNorway; ^3^ Institute of Clinical MedicineUniversity of OsloOsloNorway; ^4^ MRC Social, Genetic & Developmental Psychiatry CentreInstitute of Psychiatry, Psychology & NeuroscienceKing's CollegeLondonUK; ^5^ Norwegian Center for Child Behavioral DevelopmentOsloNorway; ^6^ Centre for Educational MeasurementUniversity of OsloOsloNorway; ^7^ Department of Special EducationUniversity of OsloOsloNorway; ^8^ Department of Child DevelopmentNorwegian Institute of Public HealthOsloNorway; ^9^ Department of Human and Molecular GeneticsVirginia Institute for Psychiatric and Behavioral GeneticsVirginia Commonwealth UniversityRichmondVAUSA; ^10^ Department of PsychiatryVirginia Commonwealth UniversityRichmondVAUSA; ^11^ School of PharmacyUniversity of OsloOsloNorway

**Keywords:** Behavior problems, Child Behavior Checklist, depression, MoBa, prenatal, postnatal

## Abstract

**Background:**

Previous studies have found significant associations between maternal prenatal and postpartum depression and child behavior problems (CBP). The present study investigates whether associations remain in a prospective, longitudinal design adjusted for familial confounding.

**Methods:**

The sample comprised 11,599 families including 17,830 siblings from the Norwegian Mother and Child Cohort study. Mothers reported depressive symptoms at gestational weeks 17 and 30, as well as 6 months, 1.5, 3, and 5 years postpartum. Fathers’ depression was measured at gestational week 17. At the last three time‐points, child internalizing and externalizing problems were concurrently assessed. We performed multilevel analyses for internalizing and externalizing problems separately, using parental depression as predictors. Analyses were repeated using a sibling comparison design to adjust for familial confounding.

**Results:**

All parental depressive time‐points were significantly and positively associated with child internalizing and externalizing problems. After sibling comparison, however, only concurrent maternal depression was significantly associated with internalizing [estimate = 2.82 (1.91–3.73, 95% CI)] and externalizing problems [estimate = 2.40 (1.56–3.23, 95% CI)]. The effect of concurrent maternal depression on internalizing problems increased with child age.

**Conclusions:**

Our findings do not support the notion that perinatal maternal depression is particularly detrimental to children's psychological development, as the most robust effects were found for maternal depression occurring during preschool years.

## Introduction

Maternal depression (MD) is associated with child behavior problems (CBP; Beck, 1999; Goodman et al., 2011), but findings on associations between perinatal MD and later CBP are mixed (Grace, Evindar, & Stewart, 2003; Waters, Hay, Simmonds, & van Goozen, 2014). Nevertheless, there exists a notion that the perinatal period is a sensitive stage for exposed children (Bagner, Pettit, Lewinsohn, & Seeley, 2010). Recently, the United States Preventive Services Task Force recommended pregnant and postpartum women to be particular targets for depression screening in the adult population (Siu & USPST, 2016). CBP are typically divided into two dimensions; internalizing and externalizing (Achenbach, 1966), characterized by negative mood states and behavioral inhibition, and by behavioral disinhibition, respectively. In general, MD, although being on the internalizing spectrum, has been found to be equally strongly associated with internalizing and externalizing problems (Goodman et al., 2011).

Maternal depression may vary with regard to timing. To be able to evaluate influences at specific phases in the child's development, it is vital to include multiple assessments of MD symptoms and CBP. Although some studies measure MD at several time‐points (e.g. Pemberton et al., 2010; Woolhouse, Gartland, Mensah, Giallo, & Brown, 2016), most are divided into those that separately investigate effects of prenatal, postpartum, or later MD. While some studies find perinatal MD to predict later CBP, even after controlling for a later or ongoing association between MD and CBP (Barker, Jaffee, Uher, & Maughan, 2011; Deave, Heron, Evans, & Emond, 2008; Verkuijl et al., 2014), others find evidence only for concurrent associations throughout early childhood (Brennan et al., 2000; Walker, Davis, Al‐Sahab, & Tamim, 2013).

Both adult depression and child internalizing and externalizing problems are heritable (Rice, Harold, & Thapar, 2002; Sullivan, Neale, & Kendler, 2000; Young, Stallings, Corley, Krauter, & Hewitt, 2000). As parents and children are genetically related and share a family environment, previously reported associations could be due to unmeasured genetic or shared environmental confounding. Several genetically informative studies have investigated associations between MD and CBP (Kerr et al., 2013; Kim‐Cohen, Moffitt, Taylor, Pawlby, & Caspi, 2005; McAdams et al., 2015; Pemberton et al., 2010; Rice, Harold, & Thapar, 2005; Silberg, Maes, & Eaves, 2010; Singh et al., 2011). With the exception of two of the studies (Kerr et al., 2013; Pemberton et al., 2010), all were based on unique samples. Associations between MD and internalizing problems are typically direct environmental (Silberg et al., 2010; Singh et al., 2011), whereas for externalizing problems, findings are more mixed, where some find evidence for genetic confounding (Kim‐Cohen et al., 2005; Silberg et al., 2010; Singh et al., 2011), some for direct environmental associations (McAdams et al., 2015), and some for both (Silberg et al., 2010). However, previous studies are subject to limitations, including small sample sizes (Kerr et al., 2013; Pemberton et al., 2010) and samples restricted to specific age groups such as toddlers (Pemberton et al., 2010) or adolescents/adults (Rice et al., 2005; Silberg et al., 2010; Singh et al., 2011), and only two previous genetically informative studies have investigated perinatal MD (Kerr et al., 2013; Pemberton et al., 2010). Some designs were also cross‐sectional (Rice et al., 2005; Silberg et al., 2010) and some were either partially (Kerr et al., 2013; Kim‐Cohen et al., 2005; Pemberton et al., 2010) or fully retrospective (Singh et al., 2011). In sum, very few studies have been both longitudinal and genetically informative. More importantly, attention has largely been focused on either perinatal depression or exposure later in development.

We address these methodological considerations by using a large longitudinal cohort sample with multiple assessments of maternal depressive symptoms (MDS) and CBP from the perinatal period through age 5. Using sibling comparison, we adjust for unmeasured genetic and shared environmental confounding, as siblings share family environment and their mother's genetic risk for depression. Prenatal paternal depressive symptoms were also included as a negative control. The aims for the study were to: (a) investigate unique associations between prenatal, postpartum, or concurrent MDS, and internalizing and externalizing problems, respectively, (b) clarify whether associations can be accounted for by familial confounding, and (c) investigate whether associations depend on child age.

## Methods

### Participants

The present study is part of a subproject of the Norwegian Mother and Child Cohort Study (MoBa), conducted by the Norwegian Institute of Public Health (NIPH). MoBa is a prospective, ongoing, pregnancy cohort study, and has previously been described in detail (Magnus et al., 2016). Participants were recruited from 1999 to 2009 at a routine ultrasound examination offered to all pregnant women in Norway at gestational week 17–18. The total sample now includes >114,500 children, >95,000 mothers, and >75,000 fathers. In total, 41% of eligible women participated. The current study is restricted to families with complete data on the time‐invariant (but not on the time‐variant) study variables, assuming Missing At Random, and more than one birth record in MoBa, encompassing 11,599 families including 17,830 full‐siblings. All included children had at least one participating, biological sibling. We use information obtained at gestation week 17 for mothers [questionnaire 1 (Q1)] and fathers (Q‐father), gestation week 30 (Q3), 6 months postpartum (Q4), and 1.5 (Q5), 3 (Q6), and 5 years (Q‐5 year) postpartum, from now on referred to as T1, T1‐father, T2, T3, T4, T5, and T6, respectively. Information was also obtained from the Medical Birth Registry of Norway (MBR; Irgens, 2000).

Version 9 of the quality‐assured MoBa data files were used, released in 2015. Written informed consent was obtained from all participants upon recruitment. The MoBa study has been granted a license from the Norwegian Data Inspectorate, and the present study was approved by the Regional Committee for Medical Research Ethics.

### Measures

#### Maternal depression

Symptoms of depression were assessed by a short form of the Symptom Checklist (SCL; Derogatis, 1994). In MoBa, the five‐item SCL‐5 is available at T1 for mothers, whereas the eight‐item SCL‐8 is included at T1‐father, and for mothers at T2 to T6. For both SCL‐5 and SCL‐8, we selected only the items intended to measure depression (three items for SCL‐5 and four for SCL‐8; Tambs & Røysamb, 2014). The participants were asked to what extent a set of statements, covering the last 2 weeks, are true on a 1 (‘not bothered’) to 4 (‘very bothered’) scale. It has previously been shown that the genetic correlation between SCL‐5 and mood disorders measured by the Composite International Diagnostic Interview was close to 1.0, suggesting that the genetic risk for depression can be captured by just five items (Gjerde et al., 2011). We calculated mean‐scores for each individual for each time‐point. The rearranging of data into long format for multilevel analyses resulted in SCL scores at T4 to T6 being represented by one time‐varying variable (SCL concurrent). Earlier SCL time‐points are separate variables as these exposures are time‐invariant for each child. Cronbach's alphas were acceptable: 0.71 at T1; 0.77 at T1‐father; 0.72 at T2; 0.77 at T3; 0.78 at T4; and 0.81 at T5 and T6.

#### Child internalizing and externalizing problems

Internalizing and externalizing problems were measured at the same age for all siblings, using items included in the Child Behavior Checklist (CBCL) for preschool children (Achenbach, 1992). In the questionnaires at T4–T6, there are in total 13 different internalizing and 11 externalizing items. For each item, mothers reported agreement using a 3‐point Likert scale: 1 = ‘not true’, 2 = ‘somewhat or sometimes true’, 3 = ‘very true or often true’. As for time‐varying maternal SCL, internalizing and externalizing problems from T4 to T6 were each represented by one variable as opposed to three. To reduce the influence of measurement error, we estimated factor scores based on an IRT analysis with a nominal response model (NRM). NRM provided better fit than a graded response model (ΔAIC = −276.7 and −211.6 for internalizing and externalizing, respectively), and was used to relax the proportional odds assumption and account for varying precision within items. For information on differential item functioning, please see Table S1 in the supplementary material, available online. The internalizing and externalizing factor scores were transformed into *T*‐scores (i.e. mean = 50; *SD* = 10).

#### Covariates

Child age was centered at 5 years (T6) and included as a continuous variable. Child sex was coded as 0 = ‘boy’ and 1 = ‘girl’. Maternal parity and education were included as these have previously been shown to associate with perinatal depression (Ohara & Swain, 1996). Parity was coded as 0 = ‘nulliparous’, 1 = ‘one previous birth’, 2 = ‘two previous births’, 3 = ‘three previous births’, and 4 = ‘4 or more previous births’. Education, coded as 1 = ‘9‐year secondary school’ through 6 = ‘University, technical college, >4 years’, was defined for the parent with the highest achieved level of education at T1 (Table 1).

**Table 1:** Variables used in the multilevel analyses

	Level	Mean	*SD*	Min.	Max.
Dependent variables					
CBCL internalizing	1	50	10	35.72	104.15
CBCL externalizing	1	50	10	31.26	92.89
Predictors (Grand‐mean centered)					
SCL 17th week of gestation	2	0	0.38	−0.22	2.78
SCL 17th week of gestation father	2	0	0.30	−0.15	2.85
SCL 30th week of gestation	2	0	0.36	−0.26	2.74
SCL 6 months postpartum	2	0	0.39	−0.26	2.74
SCL concurrent	1	0	0.43	−0.29	2.71
Predictors (Group‐mean centered)					
SCL 17th week of gestation	2	0	0.18	−1.80	2.00
SCL 17th week of gestation father	2	0	0.14	−1.50	1.69
SCL 30th week of gestation	2	0	0.16	−1.67	1.69
SCL 6 months postpartum	2	0	0.17	−1.69	2.25
SCL concurrent	1	0	0.17	−1.50	1.50
Covariates					
Child age in years	1	−2.02	1.47	−3.72	1.67
Child sex	2	0.49	0.50	0	1
Parity	2	0.79	0.82	0	4
Parental education level	2	4.91	1.04	1	6

CBCL = Child Behavior Checklist, SCL = Symptom Checklist. Predictors and covariates are grand‐mean centered lest they have a natural zero point. Group‐mean centering of predictors enables sibling comparison.

### Statistical analyses

The analyzed data follow a three‐level structure, with responses (level 1), nested in child siblings (level 2), nested in mothers (level 3). We used linear multilevel models to account for dependency across siblings within mothers (level 3) and across tests within children (level 2) by estimating between‐mother and between‐sibling differences through the inclusion of random effects (Rabe‐Hesketh & Skrondal, 2012). Maximum likelihood estimates of model parameters were obtained using Stata 14 (StataCorp, 2015). The basic model can be considered a latent growth curve model, where we allowed for a random intercept at level 2, and a random intercept and slope (a coefficient of age) at level 3. We did not allow for a random slope at level 2 as initial analyses showed that variance attributable to the effect were negligible for both outcome measures. The basic model used can be expressed as follows:

$${\text{cbcl}}_{\mathrm{ijk}}=x_{\mathrm{ijk}}^{T}\beta +\eta_{\mathrm{jk}}+\eta_{0k}+\eta_{1k}{\text{age}}_{\mathrm{ijk}}+\in_{\mathrm{ijk}}$$

where $x_{\mathrm{ijk}}^{T}$ is a transposed matrix of all included predictors, and *η*_jk_, *η*_0k_, and ϵ_ijk_ are the levels 2, 3, and 1 residual error terms, respectively, with a mean of zero and a variance to be estimated. An illustration of the model is included in Figure S1. The predictors are treated as fixed effects, whereas the error terms are random effects. Predictors from various levels can be included in the same model (Hox, 2010).

Several models extending the basic model outlined above were fitted to each of the outcome variables separately. First, to answer aim 1: what was the association between the depression variables and child CBP, and 3: do these associations vary with child age; we fitted independent models with each of the parental depression variables entered as explanatory variables (10 models). Included were adjustments for covariates and an interaction term between the depression variable and child age. The interaction acts as a predictor for the random slope across families. Second, we entered all parental depression variables simultaneously to investigate their unique effects (two models). By including fathers’ prenatal depression scores, we also have a negative control, as father's level of depression is unlikely to causally influence CBP at this time‐point. We therefore expect the regression coefficient for this predictor to be less than the maternal predictor. Third, to answer aim 2: whether associations were due to familial confounding, we proceeded with the full models from step 2, but replaced the depression variables with depression variables centered within mothers (subtracting the mother‐specific depression average across siblings from each depression score). This centering was used to obtain estimates of within‐mother effects, where unmeasured genetic and shared environmental influences that vary between mothers are removed (D'Onofrio et al., 2007).

## Results

### Internalizing problems

**Figure 1:**
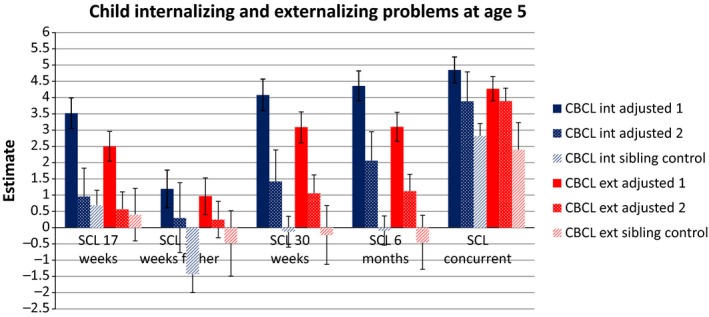
Main effects (unstandardized estimates) with 95% CI of parental depression on behavior problems at age 5. CBCL = Child Behavior Checklist; int = internalizing problems; ext = externalizing problems; SCL = self‐reported depressive symptoms from the Symptom Checklist; adjusted 1 = adjusted for covariates; adjusted 2 = adjusted for covariates + each depression time‐point; sibling comparison = adjusted for covariates, each depression time‐point and familial confounding [Colour figure can be viewed at wileyonlinelibrary.com]

Results for the full models are shown in Table 2. All independent associations (not included in Table 2, but illustrated in Figure 1) between each depression time‐point were statistically significant (*p* < .00). Adjusted for each other, all depression predictors were significant and positive, with the exception of paternal depression, and the interactions between depression at Q3 and age, and paternal depression and age. Positive interactions indicate that associations between MDS and internalizing problems continue to increase in magnitude as the child grows older. Maternal concurrent depressive symptoms had the strongest association [estimate = 3.88 (3.45, 4.31, 95% CI)], meaning that one unit increase in concurrent MDS is associated with a 3.88 units increase in internalizing problems for 5‐year‐old firstborn boys with university‐degree parents. Age had the opposite effect, where internalizing CBP are estimated to decrease .59 units each year the child grows older (95% CI = −0.65, −0.53).

**Table 2:** Results from multilevel modeling of child internalizing problems

Model	Model 1		Model 2 (sib. comparison)	
Fixed effects	Estimate (*SE*)	95% CI	Estimate (*SE*)	95% CI
Intercept	49.62 (0.14)***	49.34 to 49.89	49.53 (0.13)***	49.28 to 49.78
SCL17	0.96 (0.28)***	0.41 to 1.50	0.69 (0.44)	−0.17 to 1.56
SCL17father	0.30 (0.29)	−0.28 to 0.87	−1.43 (0.55)**	−2.50 to −0.35)
SCL30	1.42 (0.30)***	0.84 to 2.00	−0.12 (0.50)	−1.10 to 0.85
SCL6	2.06 (0.27)***	1.52 to 2.59	−0.09 (0.45)	−0.97 to 0.80
SCLconcurrent	3.88 (0.22)***	3.45 to 4.31	2.82 (0.47)***	1.91 to 3.73
SCL17 × Age	0.20 (0.10)*	0.01 to 0.38	0.30 (0.17)	−0.03 to 0.63
SCL17father × Age	0.02 (0.10)	−0.18 to 0.21	−0.33 (0.21)	−0.74 to 0.08
SCL30 × Age	−0.06 (0.10)	−0.26 to 0.14	−0.19 (0.19)	−0.56 to 0.18
SCL6 × Age	0.25 (0.10)**	0.06 to 0.43	−0.15 (0.17)	−0.49 to 0.19
SCLconc. × Age	0.64 (0.09)***	0.47 to 0.80	0.54 (0.18)**	0.19 to 0.90
Random effects	Estimate (*SE*)		Estimate (*SE*)	
*var* (ϵ_ijk_)	63.44 (0.69)	62.10 to 64.81	63.58 (0.70)	62.23 to 64.96
*var* (*η*_jk_)	8.64 (0.68)	7.41 to 10.07	8.09 (0.67)	6.87 to 9.52
*var* (*η*_0k_)	31.08 (1.30)	28.65 to 33.73	39.63 (1.42)	36.94 to 42.52
*var* (*η*_1k_)	1.06 (0.13)	0.83 to 1.35	1.18 (0.13)	0.94 to 1.47
	Estimate (corr)		Estimate (corr)	
Cov(*η*_0k_, *η*_1k_)	4.19 (0.73)	3.52 to 4.86	5.08 (0.74)	4.37 to 5.80
AIC	296,625.8		297,846.6	

Model 1 adjusted for age, sex, parity, and education. Model 2 adjusted for age, sex, and parity, and is also the sibling comparison model. SCL17 through SCLconcurrent = Symptom Checklist scores measured at 17th gestational week (T1), 17th gestational week father (T1‐father), 30th gestational week (T2), 6 months postpartum (T3) and concurrently [1.5–5 years postpartum (T4‐T6)]. *var* (*ϵ*_ijk_) = variance at the individual level, *var* (*η*_jk_) = variance in intercept accounting for dependency in repeated measures within children (level 2), *var* (*η*_0k_) = variance in intercept accounting for dependency in children within mothers (level 3), *var* (*η*_1k_) = variance in slope at mother level, Cov(*η*_0k_, *η*_1k_) = covariance between intercept and slope at mother level.

*p < .05; **p < .01; ***p < .000.

**Figure 2:**
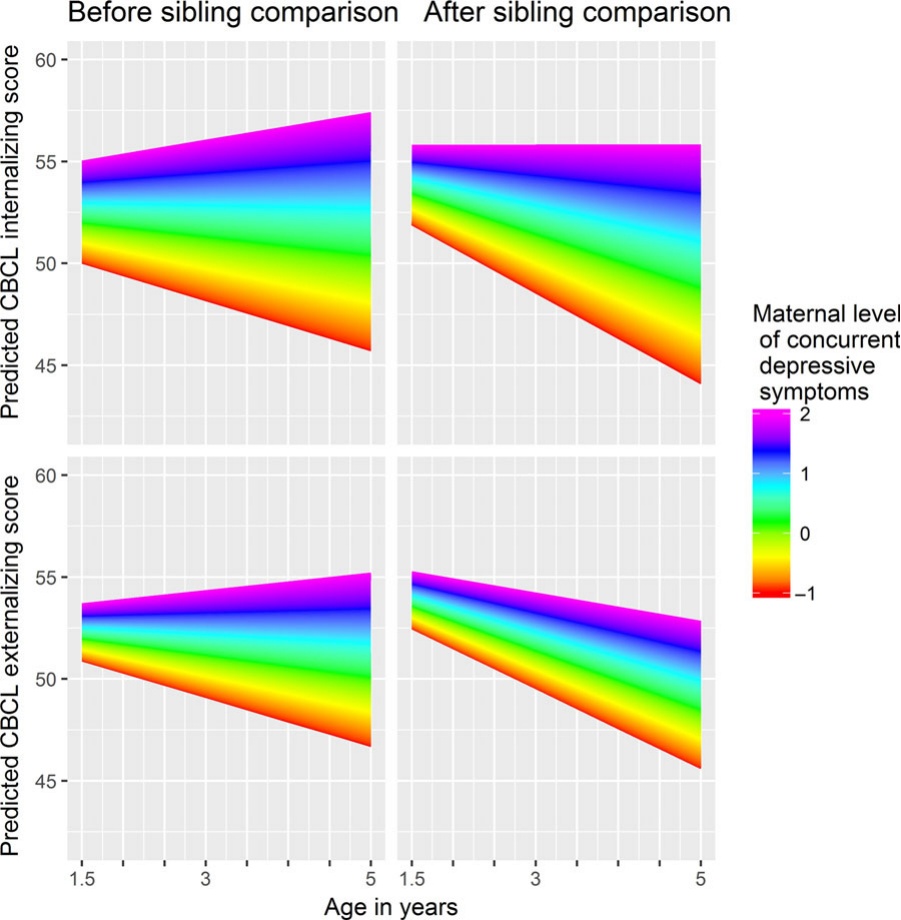
Predicted Child Behavior Checklist *T*‐scores adapted from the full models for children with mothers with high to low scores on the Symptom Checklist for concurrent depressive symptoms [Colour figure can be viewed at wileyonlinelibrary.com]

After adjusting for unobserved genetic and environmental influences at the mother level using sibling comparison (Model 2 in Table 2), most effects were attenuated, suggesting familial confounding. However, the effect of paternal depression increased. The significant depression predictors were concurrent MDS [estimate = 2.82 (1.91, 3.73, 95% CI)] and paternal depression [estimate = −1.43 (−2.50, −0.35)]. Figure 1 illustrates how the main effects change after adjusting for all depression time‐points and then familial confounding. In addition, there was a significant, positive interaction effect between concurrent MDS and child age [estimate = 0.54 (0.19, 0.90, 95% CI)], indicating increasing internalizing problems with age for children with depressed mothers (Figure 2). Children with mothers who score low on depression, however, are predicted to have a negative slope. The regression coefficient for sex remained positive, so on average, girls score .53 units higher on internalizing problems than boys.

### Externalizing problems

All independent associations were statistically significant (estimate = 0.97–4.27, *p* < .00). Figure 1 illustrates (a) independent main effects, (b) main effects after adjusting for all depression time‐points, and (c) main effects adjusted for family effects. In the full model (Table 3), all predictors and covariates were significantly associated with externalizing problems, with the exception of paternal depression, and interactions between depression from T1 to T3 and child age. Again, concurrent MDS had the strongest association [estimate = 3.89 (3.48, 4.29, 95% CI)] with externalizing problems. The earliest depression exposure had the lowest association with externalizing problems, and the effects of the depression variables increased with increasing proximity to child concurrent problems.

**Table 3:** Results from multilevel modeling of child externalizing problems

Model	Model 1		Model 2 (sib. comparison)	
Fixed effects	Estimate (*SE*)	95% CI	Estimate (*SE*)	95% CI
Intercept	48.00 (0.14)***	47.73 to 48.28	48.02 (0.13)***	47.77 to 48.27
SCL17	0.56 (0.27)*	0.04 to 1.10	0.40 (0.41)	−0.41 to 1.21
SCL17father	0.25 (0.29)	−0.31 to 0.81	−0.48 (0.51)	−1.49 to 0.52
SCL30	1.06 (0.29)***	0.50 to 1.62	−0.23 (0.46)	−1.13 to 0.68
SCL6	1.12 (0.26)***	0.61 to 1.64	−0.45 (0.42)	−1.28 to 0.38
SCLconcurrent	3.89 (0.21)***	3.48 to 4.29	2.40 (0.43)***	1.56 to 3.23
SCL17 × Age	−0.01 (0.09)	−0.19 to 0.16	−0.02 (0.15)	−0.31 to 0.28
SCL17father × Age	−0.02 (0.09)	−0.20 to 0.16	0.00 (0.19)	−0.37 to 0.37
SCL30 × Age	−0.01 (0.09)	−0.19 to 0.18	−0.05 (0.17)	−0.39 to 0.28
SCL6 × Age	−0.07 (0.09)	−0.24 to 0.10	−0.27 (0.15)	−0.57 to 0.03
SCLconc. × Age	0.74 (0.08)***	0.59 to 0.90	0.42 (0.17)**	0.10 to 0.75
Random effects	Estimate (SE)		Estimate (SE)	
*var* (ϵ_ijk_)	49.79 (0.55)	48.73 to 50.88	49.93 (0.55)	48.87 to 51.02
*var* (*η*_jk_)	12.65 (0.63)	11.47 to 13.95	12.28 (0.62)	11.11 to 13.57
*var* (*η*_0k_)	39.14 (1.33)	36.63 to 41.83	46.15 (1.43)	43.44 to 49.03
*var* (*η*_1k_)	1.28 (0.11)	1.08 to 1.53	1.36 (0.12)	1.16 to 1.61
	Estimate (corr)		Estimate (corr)	
Cov(*η*_0k_, *η*_1k_)	5.04 (0.71)	4.41 to 5.67	5.71 (0.72)	5.05 to 6.37
AIC	292,122.5		293,124.1	

Model 1 is adjusted for age, sex, parity, and education. Model 2 adjusted for age, sex, and parity. For explanation of abbreviations, please see Table 2.

*p < .05; **p < .01; ***p < .000.

When we adjusted for unmeasured confounding (Model 2 in Table 3), all effects were attenuated. Only concurrent MDS [estimate = 2.40 (1.56, 3.23, 95% CI)] and the interaction between concurrent MDS and age [estimate = 0.42 (0.10, 0.75, 95% CI)] had significant effects. Due to the strong negative effect of age, the interaction coefficient did not predict increasing externalizing problems as the child grew older (Figure 2).

## Discussion

To our knowledge, this is the first study to investigate associations between MDS and child internalizing and externalizing problems from pregnancy until 5 years postpartum that also includes rigid control for unmeasured confounding and a large sample size. Knowledge on when children are most vulnerable for developing adverse effects after exposure to MDS is crucial for preventive efforts.

Our first aim was to investigate unique main effects of MDS at varying time‐points on CBP. We found that MDS at all time‐points was uniquely and significantly associated with internalizing and externalizing problems. This fits well with previous studies that have adjusted for later depression (Barker et al., 2011; Beck, 1998; Deave et al., 2008; Verkuijl et al., 2014), although in smaller samples, associations disappear (Woolhouse et al., 2016). Conversely, paternal prenatal depressive symptoms were not found to predict CBP.

For the second and most important aim, we investigated whether associations found in the first set of analyses remained after adjusting for unmeasured familial confounding. In these analyses, only concurrent depressive symptoms had unique and adverse effects, indicating residual confounding in previous studies. We found only one adoption study to compare our results with perinatal MDS (Kerr et al., 2013; Pemberton et al., 2010). Results from this study fitted well with our findings of prenatal depression being confounded by familial factors. Most genetically informative studies using older samples (aged 9–39 years) find evidence for direct environmental effects for MD on child internalizing symptoms (Silberg et al., 2010; Singh et al., 2011) in line with our findings. The pattern also fit with those studies reporting evidence for direct environmental effects for externalizing problems (McAdams et al., 2015; Silberg et al., 2010). One potential explanation for finding effects of concurrent MD, but not MD during the perinatal period or infancy, is that depressed mothers are able to provide their children with what is needed, up until the child reaches toddlerhood. After this, the child may need more behaviorally engaged mothers. Hence, it is possible that only the risk associated with later MD is transmitted to the children. It may also take a few years before the effects are expressed as CBP. Instead, effects may be reflected in other developmental domains. For instance, postpartum MD has been found to have stronger associations with cognitive abilities than with CBP (Grace et al., 2003).

Our third aim was to investigate to what extent internalizing and externalizing problems vary over time as a function of MDS. Before the sibling comparison, there were weak associations between prenatal MDS and child age and MDS 6 months postpartum and child age and internalizing CBP. However, after the sibling comparison, only the interaction between concurrent depressive symptoms and age had a significant effect on CBP. Furthermore, the children exposed to the highest levels of concurrent MDS were predicted to develop increasing internalizing problems between age 1.5 and 5 years. As we did not include child measures before this, we cannot discard the possibility that perinatal depressive symptoms never had a transient influence. However, our results imply that children exposed to perinatal MDS do catch up with their peers’ level of CBP. Our finding of a significant interaction between concurrent MDS and age implies that screening for maternal depression in preschool years, in addition to in pregnant and postpartum women, could be beneficial.

Another test for unmeasured confounding was the inclusion of prenatal paternal depressive symptoms. As prenatal MD is assumed to have an effect on offspring through biological mechanisms (O'Connor, Monk, & Fitelson, 2014), fathers’ prenatal depression is unlikely to have a direct effect on CBP. As expected, paternal depressive symptoms did not have a unique significant effect before sibling comparison. We were therefore surprised that it had a protective effect against internalizing problems after conducting sibling comparison. It is possible that mothers with depressed spouses are more inclined to not be depressed themselves, so that the effect is really the effect of not having a depressed mother at this time‐point. Nonrandom mating is found for depression, but it is lower than for many other psychiatric disorders (Nordsletten et al., 2016). Another possible explanation could be the lack of adjustment for later paternal depressive symptoms. If we assume that fathers continue to be depressed, the protective effect could be due to fathers participating less in child rearing, perhaps isolating themselves more from the family than depressed mothers are able to do.

### Limitations

In the current study, we use mothers’ ratings of CBPs, obtained at the same time‐point as they rated their own depressive symptoms. This may have caused confounding due to shared method variance (Podsakoff, MacKenzie, Lee, & Podsakoff, 2003). When the exposure is depression, this is often referred to as sad mother bias (Grace et al., 2003), implying that depressed mothers rate children more negatively than nondepressed mothers, regardless of child symptoms. Hence, associations in the present study between MDS and CBP could either be due to a true relationship or to shared method variance. The two alternative explanations are confounded in our design, and this is a limitation in the MoBa study. One of the strengths of the current study, however, is that time‐invariant maternal rating‐bias is adjusted for in the sibling comparison analyses.

Second, there is significant attrition in MoBa, and the most severely depressed mothers may have dropped out or never participated. We therefore included all cases with data at one or more of the outcome time‐points, estimating missing data due to attrition according to the Missing At Random assumption. As multiple imputation on clustered data is still in its infancy in the statistical literature, we utilized complete data on the independent variables to achieve correct centering of the variables. Cases dropping out before 18 months were excluded, which may have introduced bias. Nevertheless, it has been shown that attrition in population‐based, longitudinal studies do not bias associations between variables (Gustavson, von Soest, Karevold, & Roysamb, 2012).

Third, we could not demonstrate invariance in item performance across the three time‐points for internalizing and externalizing problems. This may be due to our large sample size, but should nevertheless be kept in mind when interpreting the results. Differential item functioning is included in the online supplementary material.

## Conclusion

We found that only concurrent MDS had a unique and significant effect on CBP, whereas perinatal MDS appeared to be confounded by unmeasured familial factors. Importantly, the effect of concurrent MDS on internalizing problems increased with the child's age. Our findings advocate an increased focus on screening and treatment of MDS also during preschool years.

**Key points**

- Previous studies report associations between maternal perinatal depression and child behavior outcomes.
- The present study is the first to incorporate both longitudinal and genetically informative data from a large cohort sample on the unique effects of perinatal and concurrent maternal depressive symptoms on preschool child behavior problems.
- After adjusting for familial confounding, we found only concurrent maternal depressive symptoms to have an effect on internalizing and externalizing problems.
- To prevent child behavior problems, depression should be screened for and treated in mothers of preschool age children, as well as in pregnant and postpartum women.

## Supporting information


**Table S1.** Differential item functioning for the included Child Behavior Checklist items.
**Figure S1.** An illustration of the basic model used in the analyses.
