# Supplementary material for: Maternal perinatal and concurrent depressive symptoms and child behavior problems: a sibling comparison study
Source: J Child Psychol Psychiatry. 2017 Feb 23;58(7):779–86. doi: 10.1111/jcpp.12704 (PMC5484352; doi:10.1111/jcpp.12704)
Supplement: Supplementary file 1 — Table S1. Differential item functioning for the included Child Behavior Checklist items. Figure S1. An illustration of the basic model used in the analyses. [file JCPP-58-779-s001.docx]

**Supporting online information for *Maternal perinatal and concurrent depressive symptoms and child behavior problems: a sibling comparison study* *by* Gjerde et al.,**

Table S1. Differential item functioning for the included Child Behavior Checklist items

**1.5 years 3 years 5 years Pooled**

**Internalizing problems**

CBCL_10

Discrimination 2 vs 1 1.09 1.13 1.21 1.09

3 vs 1 2.46 2.33 2.49 2.37

Difficulty 2 vs 1 0.09 0.65 1.12 0.53

3 vs 1 1.64 1.84 2.20 1.85

CBCL_12

Discrimination 2 vs 1 - 0.54 - 0.52

3 vs 1 - 0.78 - 0.74

Difficulty 2 vs 1 - 3.95 - 4.19

3 vs 1 - 4.30 - 4.53

CBCL_21

Discrimination 2 vs 1 0.97 1.07 1.21 1.08

3 vs 1 2.08 1.83 1.80 1.85

Difficulty 2 vs 1 2.46 1.68 2.15 2.05

3 vs 1 3.23 2.67 2.94 2.96

CBCL_24

Discrimination 2 vs 1 0.41 0.61 0.48 0.50

3 vs 1 0.74 1.11 0.92 0.94

Difficulty 2 vs 1 3.22 1.29 2.51 2.18

3 vs 1 4.63 2.74 3.63 3.49

CBCL_33

Discrimination 2 vs 1 - - 1.11 1.15

3 vs 1 - - 2.33 2.37

Difficulty 2 vs 1 - - 0.70 0.53

3 vs 1 - - 1.86 1.70

CBCL_37

Discrimination 2 vs 1 1.10 1.12 1.45 1.12

3 vs 1 1.96 1.98 2.86 1.92

Difficulty 2 vs 1 1.31 2.01 2.06 1.76

3 vs 1 2.35 3.04 2.69 2.75

CBCL_47

Discrimination 2 vs 1 - - 2.72 2.61

3 vs 1 - - 5.02 4.82

Difficulty 2 vs 1 - - 1.83 1.73

3 vs 1 - - 2.44 2.38

CBCL_68

Discrimination 2 vs 1 - - 0.96 0.98

3 vs 1 - - 2.27 2.25

Difficulty 2 vs 1 - - 0.47 0.31

3 vs 1 - - 1.68 1.54

CBCL_78

Discrimination 2 vs 1 - 0.90 0.70 0.79

3 vs 1 - 1.52 1.57 1.47

Difficulty 2 vs 1 - 2.39 3.23 2.72

3 vs 1 - 3.23 3.63 3.38

CBCL_79

Discrimination 2 vs 1 - 0.81 - 0.78

3 vs 1 - 1.29 - 1.24

Difficulty 2 vs 1 - 1.08 - 1.16

3 vs 1 - 2.28 - 2.39

CBCL_87

Discrimination 2 vs 1 2.10 1.58 2.36 1.64

3 vs 1 2.79 4.08 4.95 3.66

Difficulty 2 vs 1 2.67 1.90 1.67 2.21

3 vs 1 3.31 2.50 2.34 2.72

CBCL_90

Discrimination 2 vs 1 - - 1.71 1.66

3 vs 1 - - 4.26 4.08

Difficulty 2 vs 1 - - 2.58 2.51

3 vs 1 - - 3.13 3.11

CBCL_93

Discrimination 2 vs 1 - 1.04 0.87 0.97

3 vs 1 - 2.10 2.22 2.05

Difficulty 2 vs 1 - 4.50 5.79 4.92

3 vs 1 - 3.77 4.36 4.03

**1.5 years 3 years 5 years Pooled**

**Externalizing problems**

CBCL_5

Discrimination 2 vs 1 1.53 1.08 1.30 1.23

3 vs 1 2.06 1.98 2.52 2.01

Difficulty 2 vs 1 0.67 0.89 1.15 0.88

3 vs 1 1.83 2.18 2.23 2.11

CBCL_6

Discrimination 2 vs 1 1.73 1.26 1.48 1.39

3 vs 1 3.13 2.37 3.02 2.63

Difficulty 2 vs 1 0.76 1.19 1.34 1.07

3 vs 1 1.68 2.11 2.08 1.97

CBCL_8

Discrimination 2 vs 1 - 1.78 2.07 1.94

3 vs 1 - 4.11 5.09 4.45

Difficulty 2 vs 1 - -0.45 0.04 -0.33

3 vs 1 - 0.76 1.09 0.81

CBCL_15

Discrimination 2 vs 1 0.69 0.98 1.16 0.88

3 vs 1 1.46 2.29 2.74 1.96

Difficulty 2 vs 1 -0.42 -1.72 0.05 -0.67

3 vs 1 1.70 -0.01 1.28 0.86

CBCL_16

Discrimination 2 vs 1 - 1.73 1.92 1.87

3 vs 1 - 3.80 4.49 4.02

Difficulty 2 vs 1 - -0.65 0.05 -0.43

3 vs 1 - 0.58 1.13 0.70

CBCL_27

Discrimination 2 vs 1 0.35 0.53 0.59 0.51

3 vs 1 0.41 0.58 0.59 0.57

Difficulty 2 vs 1 2.00 2.39 2.52 2.08

3 vs 1 5.58 5.27 5.48 4.87

CBCL_35

Discrimination 2 vs 1 0.85 1.17 1.36 1.08

3 vs 1 0.92 2.22 2.69 1.92

Difficulty 2 vs 1 2.21 0.91 1.37 1.44

3 vs 1 4.77 2.13 2.37 2.61

CBCL_40

Discrimination 2 vs 1 0.56 0.81 1.08 0.88

3 vs 1 1.32 1.94 2.46 2.00

Difficulty 2 vs 1 1.17 0.79 1.74 1.02

3 vs 1 3.09 2.54 2.75 2.52

CBCL_56

Discrimination 2 vs 1 - 0.55 0.63 0.57

3 vs 1 - 0.73 0.82 0.74

Difficulty 2 vs 1 - 5.05 4.86 4.96

3 vs 1 - 6.66 6.36 6.63

CBCL_58

Discrimination 2 vs 1 0.78 1.18 1.42 1.13

3 vs 1 0.76 1.64 1.65 1.30

Difficulty 2 vs 1 0.99 1.05 1.42 1.06

3 vs 1 3.42 2.28 2.54 2.50

CBCL_59

Discrimination 2 vs 1 1.26 0.95 1.04 1.02

3 vs 1 2.48 1.62 1.91 1.87

Difficulty 2 vs 1 -1.73 -0.69 0.55 -0.74

3 vs 1 -0.24 0.71 1.86 0.54

Figure S1. An illustration of the basic model used in the analyses


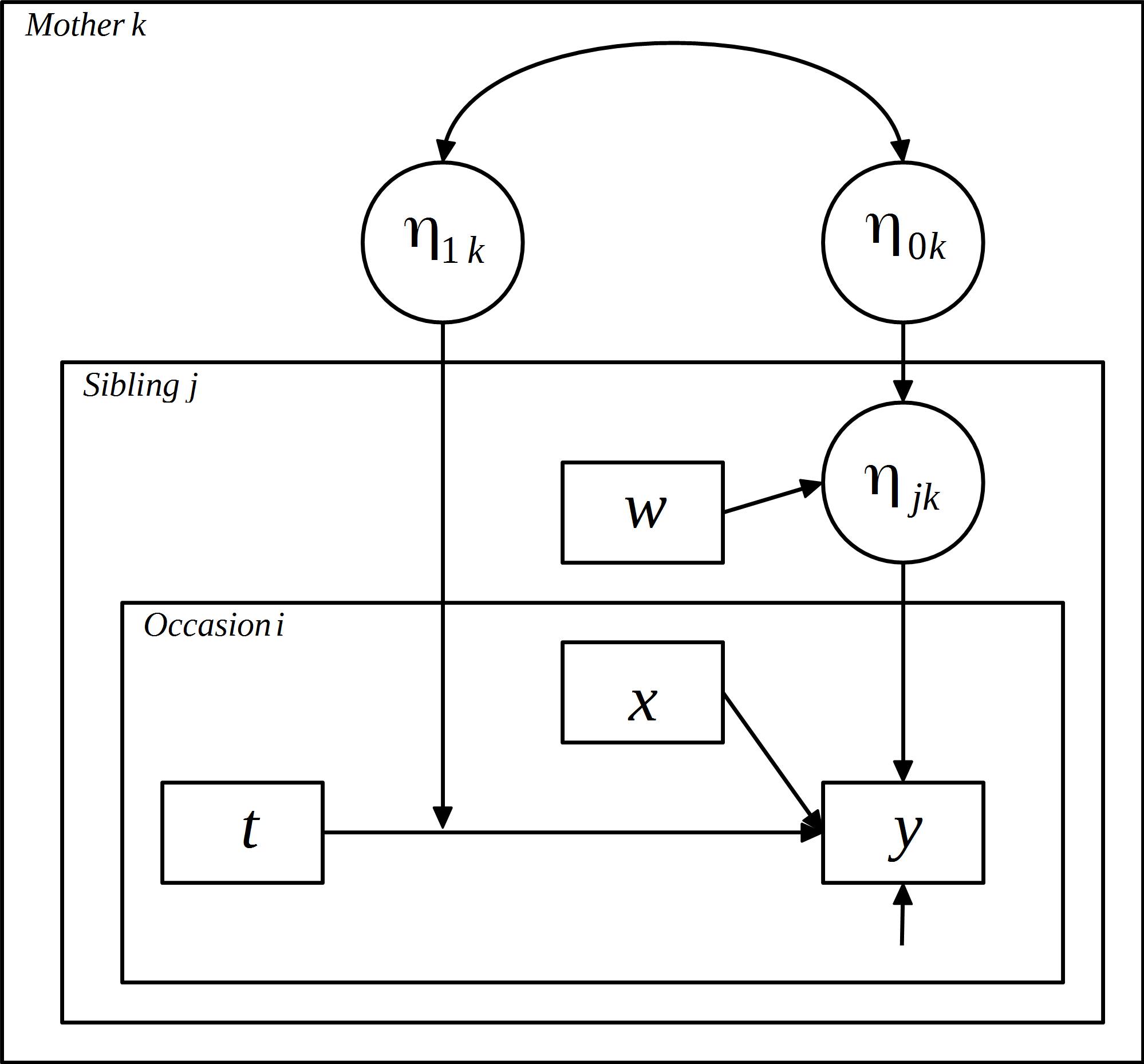


An illustration of the basic model used in the analyses. $\eta_{1k}$ = random slope at mother level; $\eta_{0k}$ = random intercept at mother level; $\eta_{jk}$ = random intercept at sibling (child) level; w = time-invariant predictor variables (parental depressive symptoms) at the child level; x = time-variant predictor variable (concurrent maternal depressive symptoms); t = time (child age). Rectangles represent fixed variables, whereas the ellipses represent latent variables. Frames represent levels of nesting. One-headed arrows represent regression effects, two-headed arrows represent co-variances.
